# Association of Online Parent‐Child Interactions With Depressive Symptoms Among Middle‐Aged and Older Empty Nesters in China

**DOI:** 10.1002/jclp.70139

**Published:** 2026-03-30

**Authors:** Wing‐yin Leung, Peiyi Lu

**Affiliations:** ^1^ Department of Social Work and Social Administration The University of Hong Kong Hong Kong China

**Keywords:** family communication, mental health, older adults

## Abstract

**Objective:**

Depression is prevalent among empty nest older adults who do not live with their children. With rapid technological development, online interaction presents a new opportunity to improve older adults' mental health. However, the relationship of online parent‐child interaction with depressive symptoms among empty nesters remains under‐examined. Guided by the social support theory, this cross‐sectional study examined the association between online parent‐child interaction and depressive symptoms among middle‐aged and older Chinese empty nesters.

**Methods:**

A total of 5198 Chinese empty nesters aged 45+ from the 2018 China Health and Retirement Longitudinal Study were included. Depressive symptoms were measured using the Center for Epidemiologic Studies Depression Scale. Participants reported the frequency they contacted their children via online. Negative binomial and Poisson regression were utilized. Interaction terms examined the moderation role of gender, marital status, and education in the relationship.

**Results:**

36.5% of participants reported elevated depressive symptoms and 59.8% of them contacted their children weekly or biweekly. More frequent online parent‐child interaction was significantly associated with lower risk of depressive symptoms (incident rate ratio = 0.981, 95% CI = 0.972, 0.990; relative risk = 0.978, 95% CI = 0.963, 0.993). The moderation role of gender and education was not significant (*p* > 0.05). However, married/partnered participants had stronger association than their partnerless counterparts.

**Conclusion:**

Online parent‐child interaction was associated with fewer depressive symptoms among Chinese empty nesters in this cross‐sectional study. These findings suggest that digital family communication may be a meaningful social context to understand their psychological well‑being.

## 1 Introduction

Population aging is accelerating globally, with those aged 65+ projected to increase from 800 million in 2024 to 2.2 billion by 2080, outnumbering children under age 18 (United Nations 2024). In China, the impact of this trend is particularly significant as it has the second‐largest population in the world. Depression is a key mental health challenge accompanying this demographic shift, impairing quality of life and increasing suicide risk. In China, 24.1% of middle‐aged and older adults aged 45+ experienced depressive symptoms (Fan et al. 2021). The prevalence is even higher among empty nesters—aging parents whose adult children no longer reside with them, at 38.6% (Zhang et al. 2020).

Currently, China has a large and growing population of empty nesters, comprising 59.7% of older adult population in 2021 (China Research Center on Aging 2024). Empty nesters are likely to experience “empty nest syndromes,” the profound feelings of sadness and loss after their children leave home, which may result in old age depression. Family relationship, particularly parent‐child relationship, is a vital source of social support that can mitigate depressive symptoms among middle‐aged and older adults.

Technological advancements have revolutionized communication methods globally, with families increasingly relying on Internet for interactions, offering new opportunities for family contact despite geographical separation. For empty nesters, online interaction may be particularly vital. Previous studies suggested online social networking may offer benefits for sustaining long‐distance family connections, as they foster a sense of belonging, shared time, and perceived closeness (Madianou and Miller 2013). In China, prior research suggested that Internet use can facilitate access to information and enhance social interaction of middle‐aged and older adults (Wen et al. 2023).

However, existing research has mixed findings on the relationship between online interaction and mental health. On one hand, several studies have found online interactions linked to lower depression risk among middle‐aged and older adults (Chai et al. 2024; Hofer and Hargittai 2024). On the other hand, excessive Internet use, to the level of addiction, may exacerbate depressive symptoms (Pham et al. 2025). Notably, Internet addiction is more commonly associated with depression among younger populations in China, whereas this pattern is less evident among older adults (Wang et al. 2013).

Given the rapid rise of technology adoption and empty nest household in China, it is crucial to investigate the relationship between online interactions and depression among empty nesters. However, to our best knowledge, this topic remained underexplored. Some studies have linked depression among empty nesters to factors such as female, widowed, limited social support and poor parent‐child relationship (Huang et al. 2020; Song et al. 2023). Other research indicated that online interactions could mitigate depressive symptoms among middle‐aged and older adults in China (Chai et al. 2024). However, these studies did not specifically examine the online parent‐child interactions or focus on empty nesters.

To address this gap, this study sought to examine the relationship between online parent‐child interactions and depressive symptoms among empty nesters in China, guided by the social support theory. Social support theory emphasizes the influence of interpersonal relationships in well‐being. Social support can positively impact well‐being through fostering regular positive emotions and experiences (Wills 1985). For older adults, adult children are a key source of such support, which can reduce depressive symptoms (Lu et al. 2023).

Online interaction tools have become accessible, requiring minimal training, which is especially beneficial for older adults to receive support remotely. Compared to other offline communication channels such as mail correspondence, online contact is low‐cost, convenient, fast, and interactive. For empty nesters, who cannot have frequent in‐person parent–child contact, regular online interactions are vital for offering emotional support across distances. Greater emotional support helps them feel closer to their family members, which in turn reduces depression (Walther et al. 2017). Additionally, older adults experience progressive physiological decline, which often leads to emerging challenges in daily living. For empty nesters, who may lack immediate familial support in their households, informational support from adult children via instant online interactions with updated advice and knowledge, becomes particularly important. These forms of online interactions may play a crucial role in mitigating feelings of depression among empty nesters by fostering support across physical distances.

Additionally, filial piety, or xiao (孝), is a fundamental value in Chinese culture that continues to play a significant role in parent‐child relationship in modern China. For Chinese empty nesters, the physical absence of their children may lead to feelings of disappointment and rejection, because their children cannot provide companionship and care, contradicting the cultural ideal of filial piety. Subsequently, the unfulfillment of filial piety is linked to mental health challenges, including depression (Wu et al. 2018). However, online parent‐child interactions may buffer these negative feelings as it allows adult children to provide support in a prompt and convenient way, partially fulfilling the filial piety expectations.

Furthermore, subgroup differences in social support and cultural expectations may further modify the relationship. Individuals in more disadvantaged status may be more inclined to seek support from adult children and hold stronger filial piety expectations, potentially deriving greater mental health benefits from online interactions. For example, research has shown women are more affected by family relationship due to traditional gender roles (Li et al. 2021). Similarly, empty nesters without a spouse or with low education may rely more on their children for support (Li et al. 2005), making online interactions particularly salient.

Based on the social support theory and filial piety, we first hypothesized a higher frequency of online parent‐child interactions would be associated with fewer depressive symptoms among middle‐aged and older empty nesters in China (Hypothesis 1). Additionally, we explored if the association differed between gender, marital status, and education subgroups. We hypothesized that empty nesters in relatively disadvantaged positions (i.e., women, partnerless, and lower education) would derive greater mental health benefits from online interactions with their adult children, as this form of contact may offer social support and align with cultural expectations. Specifically, we proposed that the association would be stronger among female empty nesters (Hypothesis 2), those who are partnerless (Hypothesis 3), and those with lower education (Hypothesis 4).

## 2 Materials and Methods

### 2.1 Data and Sample

Data from the China Health and Retirement Longitudinal Study (CHARLS) were used. CHARLS is a nationwide, population‐based longitudinal study of Chinese adults aged 45 years or older and their spouses of any age. It was designed after the Health and Retirement Study in the United States as a broad‐purposed aging and health survey in China. The sampling was selected by multistage stratified probability proportionate‐to‐size sampling from 450 villages or urban communities in 150 counties across 28 provinces in China. The CHARLS questionnaire collected extensive information on a wide range of domains, including demographic characteristics, family structure, work and retirement, income and expenditure, physical functioning and mental health.

This study used the 2018 CHARLS survey with a total of 19,816 participants. The 2018 CHARLS dataset was selected because it provided the most recent pre‐COVID‐19 data, reflecting contemporary communication patterns in China. In recent years, communication technologies have advanced rapidly, and online interaction has become increasingly common. Earlier waves (2011, 2013, 2015) were relatively outdated and might not adequately capture these recent behavioral shifts. While the latest CHARLS wave in 2020 was available, it was conducted during the COVID‐19 pandemic, which had significant effects on family interaction method (in‐person contact was banned and the interactions were changed to be remote) and older adults' mental health (e.g., feeling stressful and lonely) per China's strict quarantine policy back then.

**Figure 1:**
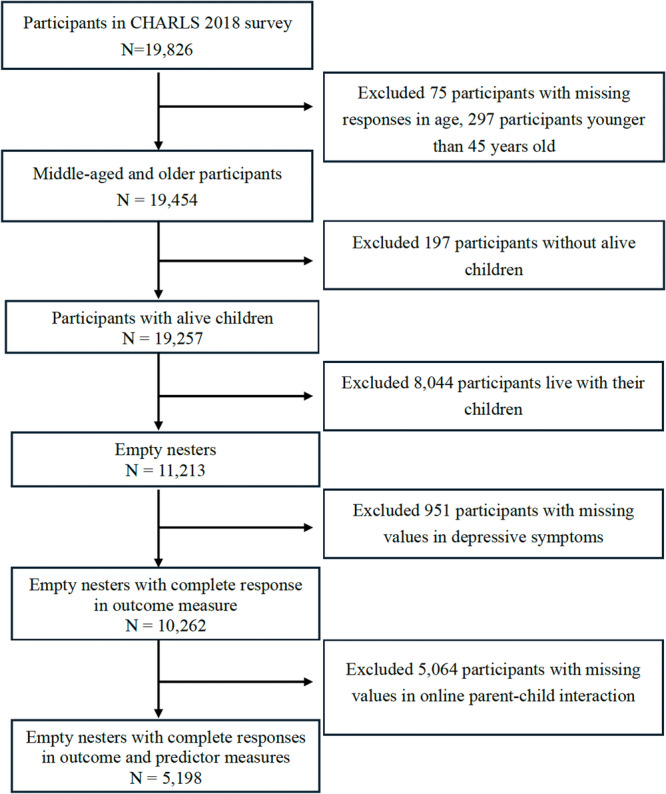
Flow chart of participant selection.

We only included participants aged 45+ who had at least one alive child and did not live with their children (i.e., empty nesters), and had complete information on both independent and dependent variables at the time of interview, resulting in a total of 5198 analytic samples (Figure 1). In this study, 99.33% of participants had adult children aged above 18 (mean age = 37.04, SD = 9.50).

### 2.2 Measures

#### 2.2.1 Independent Variable

The independent variable was the frequency of parent‐child interactions via online channels. According to CHARLS, the variable was measured by asking the participant “How often do you contact each child by phone/message/Wechat/mail/email?.” Although mailing was listed as an example, its use as an interpersonal communication method in contemporary China is likely very limited. For context, the total volume of letter correspondence in 2018 was approximately 2.67 billion items—a 15.2% drop from 2017 (Ministry of Transport of the People's Republic of China 2019). In contrast, WeChat has become the dominant mode of online interaction. A 2018 report indicated that WeChat users exchanged over 43 billion messages daily, and more than 63 million active users aged 55+ primarily engaged in video calls with their children after dinner (China News Service 2019). The indicator was measured in the number of days ranging from 1 (never) to 9 (almost every day), a higher score meant more frequent contact. If the participant had multiple children, the CHARLS survey would ask the participant how frequent each child contacted online. We created two measures to account for the presence of multiple children. First, we took the maximum value of online interactions with all children to suggest the most frequent parent‐child interactions scenario. Second, we took the mean value of online interactions with all children to suggest the average situation of parent‐child interactions.

#### 2.2.2 Dependent Variable

The dependent variable was depressive symptoms, which were self‐assessed by the 10‐item Center for Epidemiologic Studies Depression Scale (CES‐D‐10) in CHARLS questionnaire. CES‐D‐10 had adequate reliability and validity in measuring depressive symptoms in middle‐aged and older adults in China. The questions probed into the participants' feelings/experiences over the last week, such as feelings of being bothered, difficulties with concentration, depressive moods, and levels of hopefulness about the future. Each item in the CES‐D‐10 has four levels: 0 = never, 1 = sometimes or rarely, 2 = often, and 3 = always. The total score summed up all items and ranged from 0 to 30, with a higher score meaning more depressive symptoms. The depressive symptoms‐count measure counted the frequency or level of depressive symptoms, preserving the original scale's detail and enabling the retention of variability within the data. Additionally, a score of >10 was used as a cut‐off point to generate the dichotomous elevated depressive symptoms variable (1 = yes, 0 = no). The elevated depressive symptoms‐binary measure categorized individuals into two groups based on the severity of their depressive symptoms, facilitating descriptive data analysis and aiding in result interpretation.

The correlation between the count and binary measures was high (*r* = 0.837, 95% CI = 0.829, 0.845). Although related, the two measures capture different aspects of depressive symptoms: the count measure reflects symptom severity and variability, whereas the binary measure indicates clinically relevant depression risk. Using both ensures that results are robust to alternative operationalizations and remain interpretable to academic and practitioner audiences. Because dichotomization reduces information and may introduce classification artifacts, the binary measure is treated as a complementary secondary outcome, with the count measure serving as the primary indicator.

#### 2.2.3 Covariates

We selected the covariates that were likely to affect both the independent and outcome variables referring to prior research (Liu et al. 2021) and available at CHARLS study, including individual‐level demographic and health measures and household‐level characteristics. Individual‐level demographic variables included (1) age (continuous, unit: year); (2) gender (1 = female, 0 = male); (3) education (0 = no formal education, 1 = primary or middle school, 2 = high school or above); (4) marital status (1 = married/partnered, 0 = partnerless, including divorced, separated, widowed, never married); and (5) retirement status (1 = yes, 0 = no). Social activities were included as a covariate to account for potential confounding related to social connections within the neighborhood. Participants were asked whether they had engaged in any of 11 types of social activities in the past month, such as interacting with friends, attending a community club, participating in community organizations, or engaging in voluntary or charitable work (1 = yes, 0 = no). We summed responses across all items to generate a total score ranging from 0 to 11, with higher scores indicating more social activities.

Individual‐level health statuses included (1) self‐report health status (range 1 = very poor to 5 = very good); (2) number of difficulties in activity of daily living (ADL) (assessing the difficulties to perform some essential daily tasks such as eating, bathing, dressing, range 0–5, a higher value means more ADL difficulties); (3) number of difficulties in instrumental ADL (IADL, assessing the difficulties to perform some important tasks such as preparing hot meals, managing medications, range 0–6, a higher value means more IADL difficulties); (4) number of self‐reported doctor‐diagnosed chronic diseases (the history of 14 medical conditions such as high blood pressure, diabetes, range 0–14, a higher value means more chronic diseases). To account for pre‐existing depressive symptoms, we retrieved data on depressive symptoms reported by CHARLS participants in earlier waves (i.e., 2011, 2013, and 2015). Participants who reported elevated depressive symptoms in any of these waves were classified as having a history of elevated depressive symptoms; those who did not were considered to have no such history.

Household‐level measures included (1) the amount of total annual household consumption aggregated from all consumption activities (e.g., food and non‐food, unit: RMB); (2) number of alive children; (3) any children lived nearby (1 = yes, 0 = no); and (4) financial support, recorded if the participants received money from their children (1 = yes, 0 = no) or they provided money to their children (1 = yes, 0 = no).

### 2.3 Statistical Analysis

Data was analyzed with SPSS (version 29) and R software (version 4.4.2). We first described the participants' characteristics for the total sample and stratified them by depressive symptoms‐binary status. We also examined and compared the frequency of online parent‐child interactions stratified by elevated depressive symptoms‐binary status, to better understand the unadjusted relationship between independent variable and dependent variable. The t‐test and chi‐squared test was used to examine if the descriptive characteristics differ between groups with and without elevated depressive symptoms.

We then estimated separate regression models for depressive symptoms‐count and elevated depressive symptoms‐binary outcomes. By modeling the dependent variable under different distributional assumptions, we were able to assess the robustness of the observed associations across specifications. Because depressive symptoms was a count variable with high right‐skewed distribution and zero inflation, we used negative binomial regression model to estimate the depressive symptom‐count outcome as a function of covariates following prior research (Kong et al. 2024). This model examined whether online parent‐child interactions were associated with the severity or intensity of depressive symptoms across the full range of scores. Additionally, because the binary outcome variable was a common outcome (prevalence was 36.5% > 10%), we used modified Poisson regression to estimate the relative risk of elevated depressive symptoms as the logistic regression would overestimate the risk. This model focused on whether the independent variable was associated with the presence or absence of elevated depressive symptoms, aligning with mental health screening and intervention thresholds. Regression models first unadjusted and then adjusted for covariates. The statistical significance level was set at 0.05. Results were presented as incident rate ratio (IRR), relative risk (RR), and 95% confidence intervals (CI). We further computed average marginal effect (AME) to facilitate interpretation. We lastly used regression models with interaction effects to examine if the association differs by gender, marital status, and education levels. Sampling weights from CHARLS were not applied in this study because the analysis was conducted on a subset of participants and aimed to explore associations rather than estimate population‐level prevalence.

We conducted two sensitivity analyses. First, to evaluate the potential issue of reverse causality (i.e., depressive symptoms may lead to increased online parent‐child interactions), following prior research (Cho et al. 2025), we restricted the sample to individuals without pre‐existing elevated depressive symptoms in prior CHARLS waves in 2011–2015 (*n* = 2773) and reran regression models. Given that these individuals were depression‐free before entering the study in 2018, it is unlikely that their online interactions with children were motivated by elevated depressive symptoms. Second, we conducted a stratified analysis to assess whether the association differed by geographic proximity, comparing individuals who lived near their children (*n* = 955) with those who did not (*n* = 4136).

## 3 Results

### 3.1 Participants' Characteristics

The characteristics of the participants are presented in Table 1. Among the 5198 empty nesters, 50.3% were female and the mean age was 63.4 (Standard Deviation [SD] = 9.9). Majority of them were illiterate or attained low education—24% had no formal education and 64% completed primary or middle school. About 71% were married, 35% were retired, and many attended one social activity (median = 1 out of range 0–11). They reported fair self‐reported health (mean = 3 out of range 1–5, SD = 1), no difficulty in ADL or IADL, had around two chronic diseases (median = 2 out of range 0–14), and about 47% had pre‐existing depressive symptoms. Their total annual household consumption was RMB 23,078 (median). Many had two children alive and 81% did not live near their children. About 86% received financial transfers from their children and 47% of them provided financial transfers to their children.

**Table 1:** Descriptive characteristics of participants.

Variables	Levels	Total	Elevated depressive symptoms		*p*‐value
		5198	No 3299 (63.5)	Yes 1899 (36.5)	
Covariates					
*Individual‐level demographics*					
Gender, *N* (%)	Male	2581 (49.7)	1843 (55.9)	738 (38.9)	< 0.001
	Female	2617 (50.3)	1456 (44.1)	1161 (61.1)	
Age, mean ± SD		63.4 ± 9.9	63.4 ± 10.0	63.4 ± 9.6	0.05
Education, *N* (%)	No formal education	1250 (24.0)	687 (20.8)	563 (29.6)	< 0.001
	Primary or middle school	3325 (64.0)	2138 (64.8)	1187 (62.5)	
	High school or above	623 (12.0)	474 (14.4)	149 (7.8)	
Married, *N* (%)	Yes	3706 (71.3)	2441 (74.0)	1265 (66.6)	< 0.001
	No	1492 (28.7)	858 (26.0)	634 (33.4)	
Retired, *N* (%)	Yes	1804 (34.7)	1114 (33.8)	690 (36.4)	0.06
	No	3392 (65.3)	2184 (66.2)	1208 (63.6)	
Social activity, median (Q1, Q3)	0–11	1 (0, 1)	1 (0, 2)	0 (0, 1)	< 0.001
*Individual‐level health statuses*					
Self‐report health, mean ± SD	1–5	3.0 ± 1.0	3.2 ± 1.0	2.6 ± 1.0	< 0.001
ADL difficulty, median (Q1, Q3)	0–6	0 (0, 0)	0 (0, 0)	0 (0, 1)	< 0.001
IADL difficulty, median (Q1, Q3)	0–5	0 (0, 0)	0 (0, 0)	0 (0, 1)	< 0.001
Number of chronic diseases, median (Q1, Q3)	0–14	2 (1, 4)	2 (1, 3)	3 (1, 4)	< 0.001
Pre‐existing depressive symptoms	Yes	2425 (46.7)	1074 (32.6)	1351 (71.1)	< 0.001
	No	2773 (53.3)	2225 (67.4)	548 (28.9)	
*Household‐level measures*					
Household consumption, median (IQR)		23078 (12110, 40755.2)	24022 (12666.5, 42352)	21060 (11181, 38253)	< 0.001
Number of alive children, median (Q1, Q3)		2 (2, 3)	2 (2, 3)	3 (2, 3)	0.001
Lived near children, *n* (%)	Yes	955 (18.8)	608 (18.8)	347 (18.6)	0.865
	No	4136 (81.2)	2621(81.2)	1515 (81.4)	
Financial transfer from children, *n* (%)	Yes	4452 (85.6)	2806 (85.1)	1646 (86.7)	0.108
	No	746 (14.4)	493(14.9)	253 (13.3)	
Financial transfer to children, *n* (%)	Yes	2431 (46.8)	1651 (50.0)	780 (41.1)	0.000
	No	2767 (53.2)	1648 (50.0)	1119 (58.9)	
*Independent variables*					
Maximum frequency of online parent‐child interactions, median (Q1, Q3)	1–9	6 (5, 8)	6 (5, 8)	6 (4, 7)	< 0.001
Mean frequency of online parent‐child interactions, median (Q1, Q3)	1–9	7 (5, 8)	7 (5, 8)	6 (5, 7)	< 0.001

*Note:* SD = standard deviation, Q1 = 25% quantile, Q3 = 75% quantile.

When the variables were continuous measures, mean and SD were used to describe.

When the variables were count measures and highly skewed, median and Q1 and Q3 were used to describe.

When the variables were categorical, number and percentage were used to describe.

A high prevalence of elevated depressive symptoms was observed—36.5% of the participants reported CES‐D‐10 score above 10. Comparison between two groups show that those who were women, unmarried, had no formal education, fewer social activity, lower self‐report health, more ADL and more IADL difficulties, more chronic diseases, had a history of depressive symptoms, lower household consumption and no financial transfer to children, were more likely to report elevated depressive symptoms (*p *< 0.05, Table 1).

For online parent‐child interactions, the median for maximum frequency and mean frequency was 6 (every 2 weeks) and 7 (once a week), separately (Table 1). About 10% of the participants reported they contacted at least one of their children online (derived from measure of maximum frequency of online interactions) every day, 20% contacted weekly, and 16.5% contacted them monthly. However, 14% of the individuals never contacted any of their children. On average (derived from the measure of mean frequency of online interactions), 23% of the people contacted weekly, 18% contacted biweekly, and 13% contacted monthly. About 6% in general never contacted their children.

**Figure 2:**
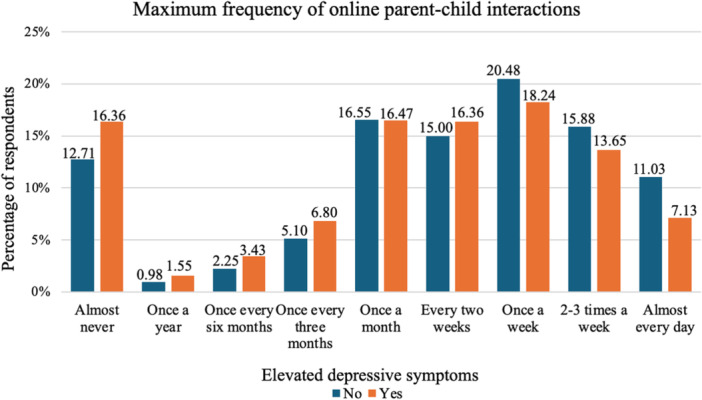
Bar plot of maximum frequency of online parent‐child interactions among participants with or without elevated depressive symptoms.

Participants with elevated depressive symptoms were found to have less frequent online interaction with their children. From the distribution of maximum frequency of online interactions (Figure 2), 16.36% of participants with elevated depressive symptoms almost never interacted with their children online compared to 12.71% of those without elevated depressive symptoms. Conversely, participants without elevated depressive symptoms were more likely to engage in frequent online interactions, with 20.48% contacting their children online weekly compared to 18.24% of those with elevated depressive symptoms.

**Figure 3:**
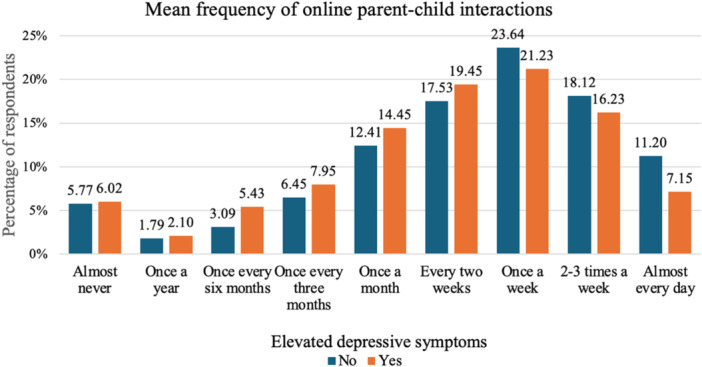
Bar plot of mean frequency of online parent‐child interactions among participants with or without elevated depressive symptoms.

Similar patterns were found in distribution of mean frequency of online interactions (Figure 3). Participants with elevated depressive symptoms generally engaged less frequently in online parent‐child interactions than those without elevated depressive symptoms. For example, 21.23% of participants with elevated symptoms interacted online weekly compared to 23.64% of those without elevated depressive symptoms. Additionally, only 7.15% of participants with elevated symptoms interacted with their children online almost every day, compared to 11.20% of those without elevated depressive symptoms.

### 3.2 Regression Results

Table 2 presents the regression model results assessing the relationship between the frequency of parent‐child online interactions and depressive symptoms among empty nesters. Most models demonstrated a significant negative association between online contact frequency and depressive symptoms. In the unadjusted negative binomial regression modeling depressive symptoms‐count outcome, more online interactions in both maximum and mean frequency measures were associated with lower risk of depressive symptoms (maximum frequency: IRR = 0.966, 95% CI [0.957, 0.975]; mean frequency: IRR = 0.965, 95% CI [0.955, 0.976]). After adjusting for covariates, the adjusted McFadden's *R*² increased; the associations attenuated but remained significant.

**Table 2:** Regression models examining the association between online parent‐child interactions and depressive symptoms.

	Depressive symptoms‐count		Elevated depressive symptoms‐binary	
	Negative binomial regression		Modified Poisson regression	
	IRR (95% CI)	AME (95% CI)	RR (95% CI)	AME (95% CI)
Model 1: Unadjusted				
*Maximum* frequency of online parent‐child interactions	0.966*** (0.957, 0.975)	−0.314*** (−0.398, −0.229)	0.954*** (0.940, 0.967)	−0.0173*** (−0.0240, −0.0106)
Adjusted McFadden's *R*²	0.047		0.049	
Model 2: Adjusted for covariates				
*Maximum* frequency of online parent‐child interaction	0.981*** (0.972, 9.990)	−0.173*** (−0.258, −0.088)	0.978* (0.963, 0.993)	−0.0082* (−0.0162, −0.0002)
Adjusted McFadden's *R*²	0.115		0.152	
Model 1: Unadjusted				
*Mean* frequency of online parent‐child interactions	0.965*** (0.955, 0.976)	−0.321*** (−0.418, −0.223)	0.955*** (0.939, 0.970)	−0.0169*** (−0.0247, −0.0092)
Adjusted McFadden's *R*²	0.020		0.021	
Model 2: Adjusted for covariates				
*Mean* frequency of online parent‐child interactions	0.985** (0.975, 0.995)	−0.139** (−0.235, −0.044)	0.984 (0.967, 1.000)	−0.0061 (−0.0151, 0.0029)
Adjusted McFadden's *R*²	0.090		0.128	

*Note:* Depressive symptoms‐count ranged 0–30 where a higher value means more depressive symptoms.

Abbreviations: AME, average marginal effect; CI, confidence interval; IRR, incident rate ratio; RR, relative risk.

Elevated depressive symptoms used the cutoff of 10 where a CES‐D‐10 score >10 was considered having elevated depressive symptoms.

Covariates included gender, age, education, marital status, retirement status, self‐report health, ADL and IADL difficulty, number of chronic diseases, total household consumption, number of alive children, living nearby children and financial transfer between children, history of elevated depressive symptoms, and social participation.

Adjusted McFadden's *R*² measures model fit while penalizing complexity. Values closer to 1 indicate better explanatory power, accounting for the number of predictors.

* *p* < 0.05

** *p* < 0.01

*** *p* < 0.001.

Similarly, in the unadjusted modified Poisson regression modeling elevated depressive symptoms‐binary outcome, more frequent online contact was associated with fewer elevated depressive symptoms, (maximum frequency: RR = 0.954, 95% CI [0.940, 0.967]; mean frequency: RR = 0.955, 95% CI [0.939, 0.970]). After adjusting for covariates, the adjusted McFadden's *R*² increased; the association between maximum frequency and elevated depressive symptoms was attenuated but remained significant (RR = 0.978, 95% CI [0.963, 0.993]). However, the association between mean frequency and elevated depressive symptoms was no longer significant (*p* > 0.05). It is important to note that the magnitude of effect was low based on the AME estimates. For example, in the adjusted models, a 1‐unit increase in maximum frequency of online parent‐child interaction is associated with an average decrease of 0.173 points in depressive symptoms‐count (95% CI: −0.258, −0.088), or a 0.82‑percentage‑point decrease in the probability of elevated depressive symptoms‐binary (95% CI: −0.0162, −0.0002), averaging over other covariates.

Regression model with interaction effects examining the moderation role of gender, marital status, and education were presented in Table 3. No significant interaction effects were observed for gender or education. However, significant interaction effects were found for marital status (*p *< 0.05). We then performed regression models separately for married/partnered and partnerless empty nesters (Table S1). Results revealed a significant negative association between online parent‐child interactions and depressive symptoms among married empty nesters, but not among partnerless individuals.

**Table 3:** Regression models with interaction effects examining the moderation role of gender, marital status and education.

	Depressive symptoms‐count	Elevated depressive symptoms‐binary
Interaction effect	Negative binomial regression	Modified Poisson regression
	IRR (95% CI)	RR (95% CI)
*Interaction with gender*		
Maximum frequency	0.987* (0.975, 0.999)	0.987 (0.961, 1.014)
Gender: male	0.938 (0.847, 1.038)	0.927 (0.734,1.168)
Maximum frequency × male	0.987 (0.971, 1.004)	0.977 (0.940, 1.016)
Mean frequency	0.995 (0.981, 1.009)	0.997 (0.967, 1.028)
Gender: male	0.997 (0.881, 1.127)	1.008 (0.760, 1.335)
Mean frequency × male	0.980* (0.961, 0.998)	0.967 (0.925, 1.011)
*Interaction with marital status*		
Maximum frequency	0.973*** (0.962, 0.984)	0.965** (0.941, 0.991)
Marital status: partnerless	0.945 (0.854, 1.046)	0.902 (0.716, 1.135)
Maximum frequency × partnerless	1.025** (1.007, 1.042)	1.034 (0.994, 1.076)
Mean frequency	0.976*** (0.964, 0.989)	0.970* (0.941, 1.000)
Marital status: partnerless	0.952 (0.842, 1.076)	0.885 (0.668, 1.169)
Mean frequency × partnerless	1.022* (1.003, 1.043)	1.036 (0.990, 1.084)
*Interaction with education*		
Maximum frequency	0.988 (0.973, 1.004)	0.986 (0.952, 1.021)
Education: primary or middle school	1.050 (0.942, 1.169)	1.090 (0.860, 1.386)
Education: High school	1.000 (0.810, 1.239)	0.952 (0.550, 1.585)
Maximum frequency × primary or middle school	0.991 (0.973, 1.010)	0.988 (0.948, 1.030)
Maximum frequency × high school	0.982 (0.950, 1.014)	0.992 (0.916, 1.078)
Mean frequency	0.998 (0.980, 1.016)	1.001 (0.963, 1.041)
Education: primary or middle school	1.086 (0.952, 1.237)	1.178 (0.881, 1.580)
Education: high school	1.169 (0.908, 1.510)	1.297 (0.671, 2.403)
Mean frequency × primary/middle school	0.985 (0.964, 1.006)	0.976 (0.931, 1.024)
Mean frequency × high school	0.959* (0.924, 0.996)	0.948 (0.862, 1.045)

*Note:* The reference level was female for gender, married/partnered for marital status, no formal education for education.

Abbreviations: CI, confidence interval; IRR, incident rate ratio; RR, relative risk.

* *p* < 0.05

** *p* < 0.01

*** *p* < 0.001.

Sensitivity analyses yielded results that were largely consistent with those from the main analyses. When restricting the sample to individuals without pre‐existing elevated depressive symptoms, the observed association remained robust, suggesting reverse causality is unlikely to pose a significant threat to the validity of our conclusions (Table S2). A significant association was observed among individuals who did not live near their children, but not among those who did (Table S3), suggesting online interaction may be especially protective for empty nesters living far away.

## 4 Discussion

To the best of our knowledge, this was the first study to investigate the association between online parent‐child interactions and depressive symptoms among empty nesters in China. A high prevalence (36.5%) of elevated depressive symptoms was observed among Chinese empty nesters. Regression results suggested that more frequent online parent‐child interactions were associated with lower risk of depressive symptoms. The direction of association was consistent across both the count and binary measures of depressive symptoms, indicating that the finding was robust to alternative operationalizations. The association did not differ across genders or education levels. However, significant association were found among married empty nesters, but not among partnerless individuals. Our finding enriched the literature on family relationships and empty nesters and suggested the potential positive role of online parent‐child interaction in mitigating old age depression.

First, elevated depressive symptoms were prevalent among Chinese empty nesters as this study found 36.5% of the participants met the criterion. This aligns closely with a previous meta‐analysis reporting that 38.6% of China empty nesters lived with depression (Zhang et al. 2020). This prevalence highlights the widespread mental health challenges faced by empty nesters who are mentally vulnerable due to physical absence of adult children, disrupted social support and traditional caregiving culture. As such, it underscores the need for targeted interventions to alleviate depressive symptoms in this population.

Next, more frequent online parent‐child interactions were associated with lower risk of depressive symptoms among empty nesters, supporting Hypothesis 1. This finding is consistent with previous research suggesting that the potential protective role of emotional and informational support provided through parent‐child interactions as explained by social support theory (Moos and Mitchell 1982; Wills 1985). Such support delivered through online communication may foster positive emotions and buffer stress among empty nesters. In China's context, online interactions may enable children to partially fulfill the responsibility of filial piety remotely, meeting empty nesters' cultural expectations. Although the association was significant, the modest effect suggests the findings should be interpreted cautiously. Our sensitivity analyses further showed the association was observed among those living far from their children, but not among those living nearby. As such, online interactions may serve as a form of social support to overcome the challenges of providing elder care from a distance and support empty nesters' psychological well‐being.

However, no significant differences were found in the association between gender or education, thus failing to support Hypotheses 2 and 4. These findings differ from the previous research that has reported gender‐ and education‐related differences in the association between parent‐child interaction and depressive symptoms (Huang et al. 2020). We postulate this might be related to the pattern of our CHARLS participants and China's cultural context. Because both genders of parents are affected by filial piety in China's context, the expectation and experience of receiving family support from adult children are shared across genders. Additionally, one possible explanation of no educational difference is that the majority of our CHARLS participants were either illiterate or had low levels of education, which limited the statistical power to detect significant differences between educational groups.

In contrast, a significant association was found among married empty nesters, but not among partnerless individuals, thereby rejecting Hypothesis 3. This finding is consistent with previous research suggesting that married parents with more frequent parent‐child interactions reported better psychological outcomes compared to partnerless parents (Pan et al. 2022). This pattern appears to extend to online interactions among empty nesters. It is possible that for partnerless empty nesters, online interactions with adult children alone may not serve as an adequate substitute for in‐person emotional support to address the deeper depressive feelings due to physical separation, especially for them without a spouse. In comparison, for married/partnered empty nesters, the combination of spousal support and online interaction with adult children may provide a synergic effect as a stronger marital bond can mutually reinforce the parent–child relationships, strengthening the positive association among married empty nesters.

This study has some notable strengths. We leveraged a large population‐based survey on nationwide sample from China to investigate depressive symptoms among empty nesters, an increasingly prevalent and urgent mental health topic in China's aging and changing societal context. Relevant covariates were considered to improve the validity of the results. Both the mean and maximum frequencies of online interactions were examined by two regression models to strengthen the reliability of the findings. We also conducted heterogeneity analyses to examine whether the association differed by some subgroups, offering insights to devise targeted intervention for subgroups.

This study also has several limitations. First, the cross‐sectional design limits the ability to establish causality. Although we conducted a sensitivity analysis to evaluate if our findings were sensitive to reverse causality, we could not completely rule out the possibility that depressive symptoms may lead to online parent‐child interactions. Longitudinal studies are needed to explore causal relationships and changes over time. The modest effect size observed in this study further constrains our ability to derive robust implications for clinical practice. Second, the reliance on self‐reported measures for interaction frequency may be subject to recall bias. While the study focused on online interactions, the raw CHARLS survey question included the mailing correspondence. Future research should aim to use measures that isolate online channels more precisely or differentiate types of communication to better understand their respective relationships with depressive symptoms. This study focused solely on the frequency of online interactions, without assessing their quality. Future research is encouraged to adopt a more comprehensive scale that captures diverse attributes of interaction. Third, a very small percentage (0.67%) of children were aged below 18, so not all study subjects were exclusively focused on relationships between adult children and aging parents. Due to data limitations, we approximated pre‐existing depressive symptoms using information from earlier waves of CHARLS. Future research should consider incorporating family history of depression prior to the onset of empty nest status to more comprehensively account for potential confounding.

Our findings provide preliminary but suggestive evidence of an association and thus have potential implications. Although the modest effect size and cross‑sectional design mean that online family contact may not be directly used as a screening tool, information about living arrangements and online communication frequency may still help clinicians and social service providers initiate discussions about family support and psychosocial stressors. In this way, online interaction patterns can serve as a contextual cue to better understand an older adult's social environment and well‐being.

Our findings may also offer tentative insights for developing supportive approaches for empty nesters. Although the evidence is preliminary, more frequent online communication with adult children could potentially complement existing sources of social support, particularly for married older adults. Improving access to user‑friendly digital platforms and offering basic training may help older adults stay connected. Encouraging adult children to maintain regular online communication when in‑person contact is difficult may also strengthen intergenerational connection. These suggestions could be viewed as exploratory directions for future work.

## 5 Conclusion

Using nationwide population‐based data from China, this cross‐sectional study found more frequent online parent‐child interactions were associated with lower risk of depressive symptoms among empty nesters. Overall, our results suggest online parent‐child interaction as a potentially relevant social context for understanding depressive symptoms among empty nesters. Future research would benefit from employing longitudinal and quasi‐experimental designs to further establish causality.

## Funding

The authors have nothing to report.

## Ethics Statement

Because this study used publicly available data with completely de‐identified information, IRB approval is not required.

## Conflicts of Interest

The authors declare no conflicts of interest.

## Supporting information


**Supplementary Table 1:** Regression models among married/partnered and partnerless subgroups. **Supplementary Table 2:** Regression models examining the association among individuals without pre‐existing elevated depressive symptoms (*n*=2,773). **Supplementary Table 3:** Regression model among living and not living near children subgroups.

## Data Availability

This study used CHARLS dataset, which is available to the public. For more information, please visit CHARLS website: https://charls.pku.edu.cn/.
