# Supplementary material for: Association of Online Parent‐Child Interactions With Depressive Symptoms Among Middle‐Aged and Older Empty Nesters in China
Source: J Clin Psychol. 2026 Mar 30;82(8):1131–41. doi: 10.1002/jclp.70139 (PMC13341020; doi:10.1002/jclp.70139)
Supplement: Supplementary file 1 — Supplementary Table 1: Regression models among married/partnered and partnerless subgroups. Supplementary Table 2: Regression models examining the association among individuals without pre‐existing elevated depressive symptoms (n=2,773). Supplementary Table 3: Regression model among living and not living near children subgroups. [file JCLP-82-1131-s001.docx]

| **Supplementary table 1**. Regression models among married/partnered and partnerless subgroups | | |
| --- | --- | --- |
|  | **Depressive symptoms-count** | **Elevated depressive symptoms-binary** |
|  | **Negative Binomial Regression** | **Modified Poisson Regression** |
|  | IRR (95%CI) | RR (95%CI) |
| ***Married/partnered participants (n=3,706)*** | | |
| Maximum frequency of parent-child online contact | 0.974***  (0.963, 0.986) | 0.966*  (0.940, 0.993) |
| Mean frequency of parent-child online contact | 0.978**  (0.964, 0.991) | 0.970  (0.941, 1.002) |
| ***Partnerless participants (n=1,492)*** | | |
| Maximum frequency of parent-child online contact | 0.995  (0.980, 1.011) | 1.000  (0.966, 1.036) |
| Mean frequency of parent-child online contact | 0.999  (0.982, 1.016) | 1.006  (0.967, 1.046) |

Note. IRR= incident risk ratio, RR= risk ratio, CI = confidence interval.

**P*<0.05, ***P*<0.01, ****P*<0.001

**Supplementary Table 2.** Regression models examining the association among individuals without pre-existing elevated depressive symptoms (n=2,773)

|  | **Depressive symptoms-count** | **Elevated depressive symptoms-binary** |
| --- | --- | --- |
|  | **Negative Binomial Regression** | **Modified Poisson Regression** |
|  | IRR (95%CI) | RR (95%CI) |
| Model 1: Unadjusted |  |  |
| *Maximum* frequency of online parent-child interactions | 0.970***  (0.956, 0.984) | 0.951**  (0.923, 0.980) |
| Adjusted McFadden's R² | 0.042 | 0.042 |
| Model 2: Adjusted for covariates |  |  |
| *Maximum* frequency of online parent-child interaction | 0.975**  (0.959, 9.992) | 0.963*  (0.931, 0.997) |
| Adjusted McFadden's R² | 0.087 | 0.109 |
| Model 1: Unadjusted |  |  |
| *Mean* frequency of online parent-child interactions | 0.973**  (0.957, 0.990) | 0.966  (0.933, 1.001) |
| Adjusted McFadden's R² | 0.020 | 0.021 |
| Model 2: Adjusted for covariates |  |  |
| *Mean* frequency of online parent-child interactions | 0.979*  (0.962, 0.997) | 0.980  (0.942, 1.020) |
| Adjusted McFadden's R² | 0.066 | 0.090 |
| Note. IRR= incident risk ratio, RR= relative risk, CI = confidence interval. | | |
| **P*<0.05, ***P*<0.01, ****P*<0.001 | | |

**Supplementary Table 3.** Regression model among living and not living near children subgroups

|  | **Depressive symptoms-count** | **Elevated depressive symptoms-binary** |
| --- | --- | --- |
|  | **Negative Binomial Regression** | **Modified Poisson Regression** |
|  | IRR (95%CI) | RR (95%CI) |
| ***Participants living near children (n=955)*** | | |
| Maximum frequency of parent-child online contact | 0.997  (0.977, 1.018) | 0.995  (0.948, 1.045) |
| Mean frequency of parent-child online contact | 1.002  (0.980, 1.025) | 1.000  (0.948, 1.056) |
| ***Participants not living near children (n=4,136)*** | | |
| Maximum frequency of parent-child online contact | 0.977***  (0.967, 0.988) | 0.974*  (0.951, 0.998) |
| Mean frequency of parent-child online contact | 0.980***  (0.969, 0.992) | 0.980  (0.954, 1.007) |

Note. IRR= incident risk ratio, RR= risk ratio, CI = confidence interval.

**P*<0.05, ***P*<0.01, ****P*<0.001
